# Lithium Toxicity and Neurologic Effects: Probable Neuroleptic Malignant Syndrome Resulting from Lithium Toxicity

**DOI:** 10.1155/2012/271858

**Published:** 2012-04-18

**Authors:** Osamede Edokpolo, Madiha Fyyaz

**Affiliations:** ^1^Department of Psychiatry, Saint Elizabeths Hospital Psychiatry Residency Training Program, 1100 Alabama Avenue, SE, Washington, DC 20032, USA; ^2^Department of Medicine, Providence Hospital Internal Medicine Residency Program, 1150 Varnum Sreet, NE, Washington, DC 20017, USA

## Abstract

*Introduction*. We present the case of a patient who developed lithium toxicity with normal therapeutic levels, as a result of pharmacokinetic interaction with Valsartan, and probable Neuroleptic Malignant Syndrome from the ensuing lithium toxicity. *Case Presentation*. A 59-year old black male with bipolar disorder maintained on lithium and fluphenazine therapy presented with a 2 week history of worsening confusion, tremor, and gait abnormality. He recently had his dose of Valsartan increased. At presentation, patient had signs of autonomic instability, he was confused, dehydrated, and had rigidity of upper extremities. Significant labs on admission were lithium level-1.2, elevated CK-6008, leukocytosis WBC-22, and renal impairment; Creatinine-4.1, BUN-35, HCO_3_-20.1, and blood glucose 145. CT/MRI brain showed old cerebral infarcts, and there was no evidence of an infective process. Lithium and fluphenazine were discontinued, his lithium levels gradually decreased, and he improved with supportive treatment including rehydration and correction of electrolyte imbalance. *Conclusions*. This case illustrates that lithium toxicity can occur within therapeutic levels, and the neurotoxic effect of lithium can include Neuroleptic Malignant Syndrome. Clinicians should be aware of the risk associated with drug interactions with lithium.

## 1. Introduction

Lithium is one of the first-line mood stabilizers in the treatment of bipolar disorder (BPD), and it has been shown to reduce the risk of suicide in patients with BPD [1, 2]. It also has other indications in psychiatry including augmentation of antidepressant therapy in depression [3]. Its use is sometimes limited by its narrow therapeutic index, thus requiring regular monitoring. Lithium levels can be affected by pharmacokinetic interactions with other drugs including angiotensin receptor blockers (ARBs) like Valsartan, diuretics, and there is also the potential for neurotoxicity including delirium, serotonin syndrome (SS), and neuroleptic malignant syndrome (NMS). We present a case of lithium toxicity with a therapeutic lithium level that we believe was the result of a pharmacokinetic interaction with Valsartan and probable NMS resulting from the ensuing lithium toxicity. 

## 2. Case Presentation

Mr. B is a 59-year-old African American male with a long history of bipolar disorder, maintained on fluphenazine decanoate, and lithium carbonate for over 30 years without any incidence of toxicity. His past medical history is significant for hypertension, Type II diabetes, and morbid obesity. He presented with a 1-week history of increasing confusion and lethargy, slurred speech, urinary incontinence, worsening tremor, and gait difficulties. He was found lying on the floor beside his bed on the day of presentation. The family was unaware of how long the patient had been lying there but found him awake and alert, unable to lift himself off the floor. About 2 weeks before presentation, his antihypertensive regimen was modified with a doubling of the dose of Valsartan to 320 mg/day, in addition to his regular lisinopril 20 mg/day. His other medications were lithium carbonate 450 mg twice daily and fluphenazine decanoate 37.5 mg every 3 weeks (last administered 3 weeks before admission). His last recorded lithium level was 0.7 just prior to the above medication changes. Other relevant baseline laboratory results a few weeks before onset of symptoms included Na-134 mEq/L, K-4.1, BUN, 23, and Cr. 2.4 mg/dL.

Collateral history from his outpatient psychiatrists revealed that patient had been stable on the above regimen of lithium and fluphenazine decanoate with no medication changes made within the past 6 months.

On examination in the ER, the patient was in moderate distress, dehydrated asking for water to drink. His vital signs were fluctuating with temperature ranging from 99.3 to 100.1, heart rate from 75 to 102, and BP from 123/69 to 223/112 all over a period of 6 hours. Cardiovascular and respiratory exams were unremarkable. Neurological exam revealed slurred speech, hand tremors, and rigidity in the extremities with decreased motor strength in the upper extremities and lower extremities. Patient was oriented to place and person but not to time.

Admission labs showed a leukocytosis of WBC-22.6, neutrophil count of 87.9%; sodium was 142 mEq/L; potassium was 3.5 mEq/L, bicarbonate 20.1 mmol/L; BUN 35 mg/dL; creatinine 4.1 mg/dL; blood glucose 145, troponin I 5.64; CPK 6008; CK (MB) isoenzyme 36.7 and a lithium level of 1.20 mEq/L. Blood, urine and sputum cultures yielded no growth. CT/MRI brain showed old frontoparietal infarct, and EEG was reported as within normal limits for drowsiness and briefly aroused state with no epileptiform discharges. CSF from spinal tap was unremarkable.

Patient was commenced on IV fluid hydration and broad-spectrum antibiotics. Lithium was discontinued and fluphenazine decanoate was not administered. Repeated blood cultures yielded no growth, and thus antibiotics were discontinued. Patient showed gradual improvement in his mental status, he had no problems with articulation, and he was fully orientated within a week of admission. His lithium levels had decreased to 0.89 on day 5 and 0.55 on day 7, and his renal function was back to baseline. Patient's vital signs stabilized, and there was minimal rigidity at the time of discharge.

## 3. Discussion

Lithium is an effective agent both in acute and prophylactic management of BPAD. It is estimated that close to 80% of patients with mania respond to lithium therapy in the acute phase. It is known that lithium has a narrow therapeutic index, and it has been reported that over 75% of patients on long-term lithium therapy will experience some form of toxicity [4]. Many of these cases are the result of pharmacokinetic interactions with other drugs including ARBS, NSAIDS, and diuretics. Dehydration and drug overdoses are other causes of lithium toxicity.

Our patient had features consistent with lithium toxicity; confusion and lethargy, slurred speech, urinary incontinence, worsening tremor, and gait difficulties. Typically signs of Li toxicity are evident with high lithium level greater than 1.5 mEq/L [5], but there have been case reports of toxicity even within therapeutic levels [6, 7]. Our patient had a level that was the upper limit of normal; however, considering that there was a 70% increase in his lithium levels shortly after the increase in the dose of Valsartan makes a very strong case for lithium toxicity. We believe this was as a result of pharmacokinetic interaction between lithium and ARB, and this is consistent with previous case reports of similar interactions [8, 9].

The other consideration in our patient was NMS. This is a rare but potentially life-threatening condition associated with the use of psychotropic medications. Incidence is 0.01-0.02% in patients treated with antipsychotics [10]. While the pathogenesis of NMS is not completely understood, the generally accepted mechanism is that of widespread block of dopaminergic activity in the brain [11]. Consistent with this is the finding that the propensity for NMS from antipsychotics parallels their dopamine blockade, the development of NMS in Parkinson disease patients following abrupt withdrawal of dopamine medications, and the effectiveness of dopamine enhancing medications, for example, bromocriptine in the treatment of NMS. Further supporting the hypothesis of dopaminergic hypoactivity in the pathophysiology of NMS is the finding of low levels of the dopamine metabolite homovanillic acid in patients with acute NMS [12]. The characteristic features of this condition include fever, muscular rigidity, autonomic instability, and altered mentation. DSM-IV-TR requires muscle rigidity and fever to be present for the diagnosis [13]. It also lists other features as tremors, incontinence, dysphagia, elevated creatinine kinase, and leukocytosis. Our patient had most of the above-listed features, which makes us to postulate that he probably had NMS. If this were the case, this would be an atypical presentation given that he had been on same dose of lithium and fluphenazine decanoate for many years. It is believed that 66% of cases occur within the first week, and virtually all cases within 30 days [14]. He had risk factors for NMS including radiological evidence of cerebrovascular disease [15], been on high potency neuroleptic medication [16], and been a male. The question then is could he have developed NMS as a result of lithium toxicity? There have been many case reports of NMS resulting from Lithium and antipsychotic combination [17–20] but none has been this late in treatment or directly ascribed to lithium levels. We are aware of only 2 case reports in the literature in which lithium was believed to have led to the development of NMS independent of other neuroleptic agents [21, 22], and this could have been the case in our patient.

NMS is usually self-limiting as soon as the offending agent is withdrawn with over 60% of patients recovering within one week [14]. The mainstay of treatment of NMS remains supportive measures including hydration, correction of electrolyte imbalance, temperature control, and treating any coexisting infection.

## 4. Conclusion

Our case highlights a few important points. Lithium toxicity results from a pharmacokinetic interaction with ARB, the potential for developing toxicity even with therapeutic lithium level is noted, and the difficulty in distinguishing lithium toxicity from NMS. We wonder if the elevated lithium level was the trigger for the development of NMS in this patient given that he had been on a combination of lithium and fluphenazine for over 2 decades without any problems.

Finally while our patient made a quick recovery, the potential for syndrome of irreversible lithium effectuated neurotoxicity (SILENT) [23] should not be lost on clinicians particularly when combined with antipsychotics. Psychiatrists should also be very mindful of the nature of drug interactions particularly in patients on medications with very narrow therapeutic index like lithium, and regular liaison with the primary care physicians is suggested.
